# Coercively Adjusted Auto Regression Model for Forecasting in Epilepsy EEG

**DOI:** 10.1155/2013/545613

**Published:** 2013-04-28

**Authors:** Sun-Hee Kim, Christos Faloutsos, Hyung-Jeong Yang

**Affiliations:** ^1^Department of Computer Science, Carnegie Mellon University, Pittsburgh, PA 15213, USA; ^2^Department of Computer Science, Chonnam National University, Gwangju 500-757, Republic of Korea

## Abstract

Recently, data with complex characteristics such as epilepsy electroencephalography (EEG) time series has emerged. Epilepsy EEG data has special characteristics including nonlinearity, nonnormality, and nonperiodicity. Therefore, it is important to find a suitable forecasting method that covers these special characteristics. In this paper, we propose a coercively adjusted autoregression (CA-AR) method that forecasts future values from a multivariable epilepsy EEG time series. We use the technique of random coefficients, which forcefully adjusts the coefficients with −1 and 1. The fractal dimension is used to determine the order of the CA-AR model. We applied the CA-AR method reflecting special characteristics of data to forecast the future value of epilepsy EEG data. Experimental results show that when compared to previous methods, the proposed method can forecast faster and accurately.

## 1. Introduction

Forecasting time series data predicts future values by discovering a set of rules or identifying patterns from past data. Linear regression models for forecasting time series such as autoregressive (AR), moving average (MA), and autoregressive moving average (ARMA) are widely used [1]. However, these methods have difficulty obtaining accurate forecasts when the time series data has nonlinear characteristics that constantly change. Thus, soft computing techniques, such as fuzzy logic and neural networks, have been developed to resolve the problems of linear approach considering nonlinear properties and uncertainty of time series data [2, 3].

EEG time series signals obtained from a brain have irregular and complex wave structures. They also include a large amount of noise. Epilepsy EEG data is a representative example of a complex time series. Epilepsy is a disease defined by abnormal electrical activity in the brain that is central to the diagnosis of epilepsy. Epilepsy EEG signals display changes over time through constant interaction with external factors [4]. Noise is included within the complexity of epilepsy EEG data during measurements. Epilepsy EEG data is difficult to forecast because it has special characteristics, such as nonlinearity, abnormalities, and noise. Therefore, it is important to select an appropriate forecasting method because these characteristics affect the forecasting accuracy. 

In recent years, studies have been conducted to automatically detect and predict epilepsy seizures using EEG data. Univariate, bivariate, and multivariate algorithms were proposed to solve the problem of seizure detection and prediction based on the EEG analysis of single or multiple electrodes [5–7]. Rabbi et al. applied nonlinear dynamics based on unvaried characteristic measurements to extract a correlation dimension from the intracranial EEG recordings and designed a fuzzy rule-based system for seizure prediction [8]. Iasemidis et al. proposed an adaptive seizure prediction algorithm (ASPA) based on the convergence of the short-term maximum Lyapunov exponents (STLmax) among critical electrodes in the preseizure phase [9]. Liu et al. introduced a seizure prediction approach using particle filtering [10]. Also, Shahidi Zandi et al. proposed a method to predict seizures by analyzing the entropy level corresponding to zero-crossing intervals in scalp EEG and its derivatives [11]. Many researchers also used autoregressive and spectral analysis for forecasting by extracting seizure precursors from the EEG data [12, 13]. However, these researchers used approaches that were based on the linear data or unvaried characteristics of data.

We can give the following problem definition.

*Problem Definition.* Given a time series data which includes special characteristics such as nonlinearity, nonnormality, and nonperiodicity, a forecasting model attempts to forecast the values over some future time period. More formally, given a time series of Epilepsy EEG *X* = *x*_1_, *x*_2_,…, *x*_*t*_, we forecast the value of *x*_*t*+*n*_. *x*_*t*_ is the value of the time series at time *t*, and *n* is the forecasting length.

In this paper, we propose an adaptive forecasting algorithm that adjusts its coefficients of the autoregressive (AR) model forcedly. To forecast the future values of epilepsy EEG data including special characteristics, we use the random coefficients with −1 and 1 and the fractal dimension which the order of the CA-AR model determines. We conduct experiments with sets of EEG time series to evaluate the suitability of our forecasting approach. The experimental results demonstrate that the proposed method provides better forecasting performance than previous methods. The proposed algorithm provides the following benefits: (1) seizure forecasting and warning to patients about seizures and (2) actively probing the characteristics of seizure onset. 

The remainder of the paper is organized as follows. In Section 2, we describe the proposed method for forecasting. The experiment results are compared with the other methods in Section 3. In Section 4, we discuss the related work on the prediction and analysis of seizures. Finally, we present our conclusions in Section 5.

## 2. Materials and Methods

An autoregressive model is a simple model to estimate the future value of a series using previous input values. The AR(*p*) model represents a stochastic process using a general form of order *p* as shown in (1):

(1) Xt=c+φ1Xt−1+φ2Xt−2+⋯+φpXt−p+εt,Xt=c+∑i=1pφiXt−i+εt,

where *X*_*t*_ is the observation at time *t*,  *φ*_*p*_ is the autoregressive coefficient of *p*th order, and *ε*_*t*_ is white noise normally distributed with mean zero and variance *σ* ^2^ at time *t*. 

An important feature of the AR model is utilizing recent past observations in the process of estimating the current observation *X*_*t*_ at time *t*. That is, the current observation can be estimated by a linear weighted sum of previous observations. The weights denote the auto-regression coefficients. The problem in AR analysis is the assumption that data is stationary and linear, and it must derive the best values for coefficients given a series *X*_*t*_. Several methods have been used to estimate AR parameters, such as Yule-Walker, least squares, and Burg's method [1]. It has been shown that for large data samples these estimation techniques should lead to approximately the same parameter values. The Yule-Walker method applied the AR model to the signals by minimizing the forward forecasting error in a least squares sense. The unknown parameter *φ* is estimated as follows:

(2) φ^=∑i=1pXtXt−i∑i=1pXt−i2.

Under the assumption that *ε*_*t*_ is normally distributed, this is also the maximum likelihood estimate of *φ*. The distribution of $\hat{\varphi }$ has been studied extensively. Unfortunately, the exact distribution of $\hat{\varphi }$ is unknown. Asymptotically, if |*φ*| < 1, it has a normal distribution, while if |*φ*| > 1, it is a Cauchy distribution. In addition, if |*φ*| = 1, it is a nonstandard distribution [14]. These distributions can be used to approximate the finite sample distribution of $\hat{\varphi }$. This suggests that the distribution would not adequately approximate the finite sample distribution, especially near the discontinuity point of *φ* = 1, because the exact distribution of $\hat{\varphi }$ is continuous for all values of *φ*. It has been found that, unless *φ* is close to zero, these distributions do not approximate the distribution of finite samples well. The nonstandard limiting distribution when |*φ*| = 1 seems to give a good approximation of the finite sample distribution when |*φ*| is close to 1. However, it is too complex for practical use since an accurate approximation to this nonstandard limiting distribution can be obtained from the asymptotic expansion [15]. In this study, our aim is to find a forecasting method suitable for epilepsy EEG data. More specifically, suppose that {*φ*_*t*_} is an independent and identically distributed sequence defined by

(3) φt={θwith  probability  α,−θwith  probability  1−α,

where 0 ≤ *θ* < *∞* and 0 < *α* < 1. From (3), we have *φ* = *E*(*φ*_*t*_) = 2(*α* − 1)*θ*,  *E*(*φ*_*t*_^2^) = *θ* ^2^, and *σ*_*φ*_^2^ = *θ* ^2^ − *φ* ^2^ = 4*α*(1 − *α*)*θ* ^2^ [16].

In particular, if we take *θ* = *p*, we obtain a special case of an AR(*p*) process [16]. We study this special case in this paper. As a motivation for the model in (3) for {*φ*_*t*_} with *θ* = *p*, consider the order of the standard flexible coefficient AR(*p*) model given in (1). If *φ* = 1, we have (4) leading to the standard random model from (1):

(4) Xt(p)=∑i=1t−pεt−i.

If we put *φ* = − 1, we have

(5) Xt(p)=∑i=1t−p−(1)iεt−i.

Both models (4) and (5) correspond to the standard (critical case) unit root [17]. A model that generalizes (4) and (5) is

(6) Xt=∑i=1t−pξtiεt−i,

where the random coefficients {*ξ*_*ti*_} take the values 1 or −1. The model in (6) can be viewed as a generalization of the standard random where the successive jumps go up or down according as *ξ*_*ti*_ = 1 or −1.

Epilepsy EEG data has special characteristics, such as nonlinearity and abnormal and nonstandard distributions [4]. Therefore, in this study, when EEG data has an abnormal distribution, we forcefully adjust the coefficients of AR. In this paper, the AR model is the basis of our coercively adjusted AR model (CA-AR). This model can be expressed as follows:

(7) Xt=c+∑i=1pπtp·Xt−i+εt,

where *π*_*tp*_ is random coefficients with *φ* = 1 and *φ* = −1, with *π*_*t*1_ = 1 and |*π*_*tp*_ | = 1 for all *t* and *p*. *p* has an important role in AR modeling since it determines the order of the coefficients. In the autoregressive model, to determine the order of the AR model is an important issue [18]. The order of an AR model, *p*, must be appropriately selected because it determines the efficiency of the autoregressive model. If *p* is smaller, then the estimation error is higher while calculation speed is faster. On the other hand, if *p* is bigger, there are drawbacks requiring more computation time without any decreases in estimation error. Therefore, in order to resolve these drawbacks, an optimal way to determine the order of the AR model is required.

In this paper, the fractal dimension is used to determine the order of the CA-AR model. To calculate the fractal dimension, we apply the box-counting method [19]. The box-counting method is one of the most common methods to obtain the fractal dimensions using boxes that are big enough to cover the measured signal *S* [20]. In other words, when the length of one side of the square is *ε* (*ε* > 0) and the number of square boxes is *N*_*ε*_, the box-counting dimension of *S* is *N*_*ε*_(*S*) ~ 1/*ε* ^*d*^ and *ε* → 0. It can be expressed as the box-counting dimension of *S*,  *d*, and the positive constant, *k*:

(8) lim⁡ε→0Nε(S)1/εd=k.

By taking logs on both sides of (8), we get

(9) lim⁡ε→0(ln⁡Nε(S)+dln⁡ε)=ln⁡k.

Fractal dimension *d* is given by (10):

(10) d=lim⁡ε→0ln⁡k−ln⁡Nε(S)ln⁡ε=−lim⁡ε→0ln⁡Nε(S)ln⁡ε,

where ln⁡*k* is excluded while the denominator *ε* → 0. Also, 0 < *ε* < 1, and if ln⁡*ε* is a negative number, *d* will be a positive number. If the log-diagram of ln⁡*ε* verses ln⁡*N*_*ε*_ is a straight line, the fractal dimension is the slope of this straight line (as shown in Figure 3). 

In this paper, the measured value *d* using (10) is defined as the order of AR, *p*, and it is applied to the CA-AR(*p*) model. In other words, to predict epileptic seizures from EEG data, the future value *X*_*t*_ is predicted using *X*_*t*−1_,…, *X*_*t*−*p*_, the observed values from the past. This paper will show that special case of epileptic seizure where *X*_*t*_ is predicted by *X*_*t*−1_,…, *X*_*t*−*p*_, where the series *X*_*t*_ is AR(*p*) using (7). The estimated AR model is then used for prediction by applying the least squares estimation. 

## 3. Results 

In this section we present the empirical verification of our data analysis to forecast epilepsy EEG data. EEG datasets are provided in [4], and epilepsy EEG data set composed the five EEG datasets 5 (denoted by A~E). A and B datasets are recorded in the relaxed awake state of healthy volunteers (eye open or closed). C and D are measured during seizure free intervals, and E contained seizure activity. These five EEG datasets contain 100 single channel EEG segments of 23.6 sec duration, and they are sampled at 173.61 Hz. For our experiments, we used only three datasets such as A, C, and E. 

### 3.1. Detection of Special Characteristics

**Figure 1:**
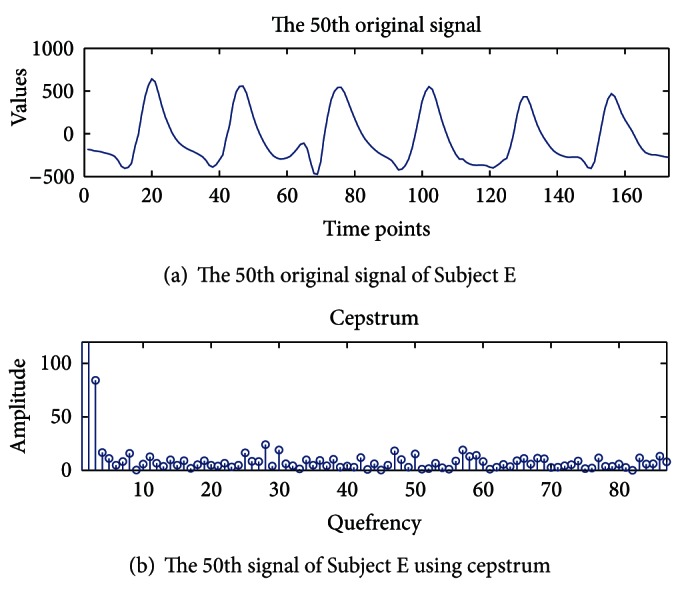
Periodicity detection using cepstrum in Subject E.

In this paper, we proposed a novel approach to help in the improvement of epileptic seizure forecasting in nonlinear and nonperiodic EEG signals. In this section, we first analyze the characteristics of epilepsy EEG data which show nonlinearity and periodicity by applying cepstrum and lag plots. The cepstrum is employed to extract periodicities or repeated patterns [21]. The cepstrum analysis of a spectrum will have peaks corresponding to the spacing of the harmonics and sidebands. The *x*-axis of the cepstrum shows frequency, and peaks in the cepstrum are related to periodicities. The cepstrum is employed to find the periodicity in Subjects A, C, and E.  Figure 1(a) demonstrates the 50th original signal of Subject E recorded during seizure activity and Figure 1(b) displays the measured periodicity using the cepstrum.  Figure 1(a) seems to have a periodic wave within the original signal. However, Figure 1(b) does not have any periodicity. In addition, Subjects A and C also do not show the periodicity.

Even though the periodicities in the original signal repeatedly appear as a sinusoidal wave during seizure activity, when we applied the cepstrum to the seizure activity signals, the results differ from the original signal. Seizure activity signals do not have any periodicity. We observed that our experimental results of the seizure activity signals by the cepstrum do not have any periodicity. Therefore, since most conventional forecasting or prediction approaches require periodicity in observed data, these approaches are not appropriate for the nonperiodic seizure activity signals. 

**Figure 2:**
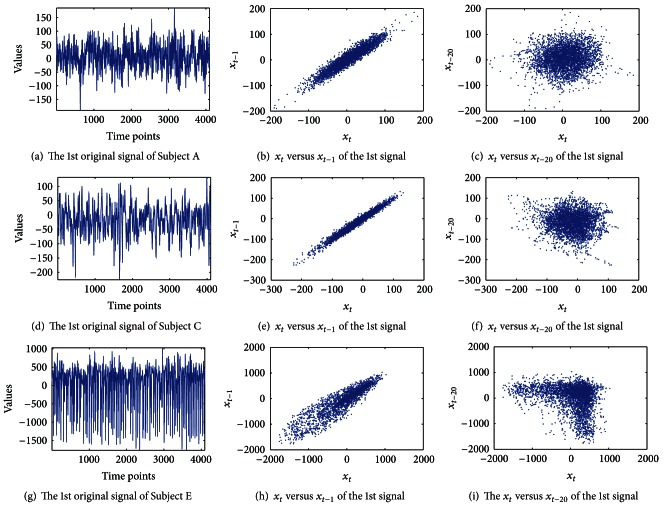
Lag plots: epilepsy EEG dataset. Subject A is shown in (a). This appears hard to predict. (d) is Subject C, and Subject E is shown in (g). (b), (e), and (h) show the two-dimensional lag plots of *x*_*t*_ versus *x*_*t*−1_, for each subject. (c), (f), and (i) are lag plots of *x*_*t*_ versus *x*_*t*−20_, respectively.

In this paper we also applied lag plots to find hidden characteristics in the data. Lag plots are useful in the analysis of cyclical data [22]. A lag plot checks whether a dataset is random or not. In addition, they provide the autocorrelation of the data.  Figure 2(a) shows the first raw signal of Subject A's epilepsy EEG time series. The lag plot of Subject A is shown in Figures 2(b) and 2(c), where the lag *L* = 1 and *L* = 20, respectively.  Figure 2(b) shows a definite linear structure in the lag plot, which was hidden in Figure 2(a). That is, this lag plot exhibits a linear pattern. If the data is strongly nonrandom, we are able to apply an autoregressive model that might be appropriate for prediction.  Figure 2(c) shows the Gaussian distribution of Subject A plotted with a lag of *L* = 20 by plotting *x*_*t*_ versus *x*_*t*−20_.  Figure 2(d) is the first raw signal of Subject C. Figures 2(e) and 2(f) show lag plots for *L* = 1 and *L* = 20, respectively. In the case of *L* = 1, linear patterns are shown for both Subject A and Subject C.  Figure 2(f) shows similar results to Figure 2(c). However, Figures 2(h) and 2(i) for Subject E differ from Subject A and Subject C. In the case of *L* = 20 shown in Figure 2(i), it is a mixture of Gaussian distribution. Generally, when a lag plot has a nonrandom pattern, the data can be predicted using conventional methods. However, the epilepsy EEG dataset shows random patterns that are difficult to be predicted by conventional approaches. Thus, we need a suitable method for forecasting of EEG signals whose characteristics are nonlinearity and nonperiodicity.

### 3.2. The Order of CA-AR Choice (Box-Counting) for Forecasting

In this section, we present how the order of CA-AR is determined using fractal dimension. We use box-counting analysis which is a common method for fractal dimension estimation. It is also known that it is easy, automatically computable, and applicable to patterns with or without self-similarity [20]. However, this technique, including the processing of data and definition of the range of grid size, requires proper implementation to be effective in practice. In this study, the grid size is changed from 0.1 to 10000 in multiplication of 2 [22]. The slope of the linear part of the plot is the estimated fractal dimension *d* of the epilepsy dataset. In this method each signal is covered by a sequence of grids of ascending sizes. Two values are recorded for each of the grids: the number of square boxes intersected by the signal, *N*_*ε*_(*S*), and the side-length of the squares, *ε*. The slope *d* of the straight line formed by plotting log⁡⁡(*N*_*ε*_(*S*)) against log⁡(1/*ε*) indicates the degree of complexity or fractal dimension between 1 and 2 (1 ≤ *d* ≤ 2) [23]. A signal with a fractal dimension of 1 or 2 is considered as completely differentiable or very rough and irregular, respectively. 

**Figure 3:**
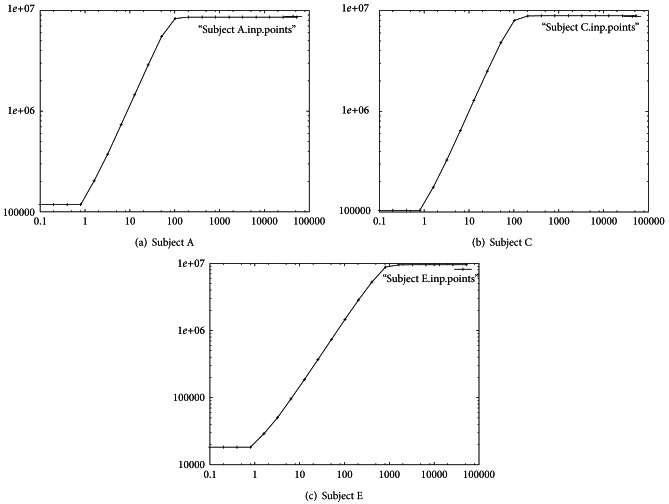
Fractal dimension: the log-log plot about the 1st signal (a vector *x*_*t*_) for each of Subjects A, C, and E.

We measured the fractal dimension of the 100 single signals from each subject to determine the order of CA-AR using box-counting analysis. Figure 3 illustrates the plot of log⁡⁡(*N*_*ε*_(*S*)) versus log⁡(1/*ε*) based on the grid sizes. The log-log plots of Figure 3 are used to estimate the fractal dimension that is computed from the slope of the plot. Figures 3(a), 3(b), and 3(c) are the log-log plot for the fractal dimension of the 1st signals of Subject A, Subject C, and Subject E. This graph clearly displays a horizontal straight line when the grid size is small or too big. Deviation from a linear straight line can be expected to lead to underestimation of the fractal dimension value for the skeleton of a signal.

We applied the box-counting method to estimate the fractal dimension of the Phase Space from a signal of each subject. The vector space of the delay coordinate vectors is termed the Phase Space [22]. The observation sequence is represented by the series *x*_*t*_, which gives the value of the time series at time *t*. That is, we can define *V* = [*x*_*t*_, *x*_*t*−*τ*_, *x*_*t*−2*τ*_,…, *x*_*t*−*Lτ*_]. *τ* is a real number greater than zero termed the time delay, and *L* is any integer greater than zero. The vector *V* is termed the delay coordinate vector, because its terms are the time-delayed data values from the time series. Given time series *x*_*t*_ and lag *L*, we form all the delay coordinate vectors from *x*_*t*_. The Phase Space is a (*L* + 1)-dimensional space. 

**Figure 4:**
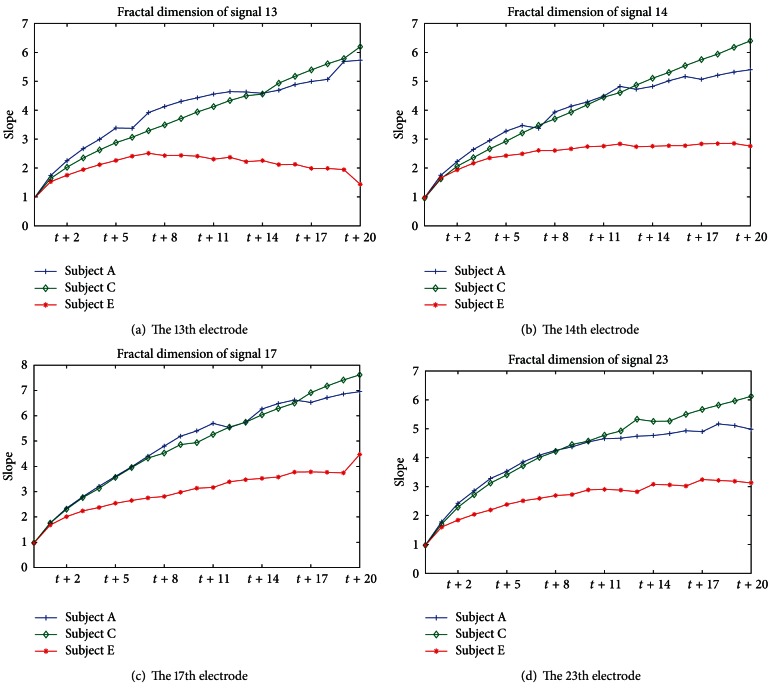
Fractal dimension of time delay space: (a) the 13th electrode of each subject, (b) the 14th electrode, (c) 17th electrode, and (d) 23rd electrode.

Figure 4 demonstrates the estimated fractal dimensions. The *x*-axis and *y*-axis denote the Phase Space (time delay space) and slope, respectively. This implies that a lag length of one is sufficient to reconstruct the state space. 

Figure 4 shows fractal dimension of few signal, and we confirm some particular results; if the dimension of signal *x*_*t*_ increases, the fractal dimension (slope) increases. As a result of Figure 4, in the case of Subjects A and C, signals mostly have a fractal dimension between 5 and 7, while Subject E exhibits a fractal dimension between 2 and 4 when time delay is 20. That is, Subjects A and C exhibit an average slope between 4 and 5. However, in the case of Subject E, the seizure activity represents an average slope of 2.5. When the time delay dimension increases, the fractal dimension increases. However, the plot of fractal dimension versus lag length shows that fractal dimension does not significantly increase, as lag length is incremented. This experiment shows the determination of the value of parameters using log-log plots for time series prediction. That is, we select the round-up integer of the average slope values of all normal signals as Subjects A and C for the order *p* of CA-AR, because we must predict abnormal behavior that dropped out of the past pattern. We ran the CA-AR model with the round-up integer “5” of the average of the slope of all signals for forecasting. To verify that the selected *p* = 5 is the optimal order, we measured the forecasting error of each signal from each subject. That result can be confirmed in Section 3.3.

### 3.3. Forecast Accuracy

To evaluate the reliability of optimal order for our model, we measured Root Mean Square Error (RMSE) of forecasting from all signals of each subject. An autoregressive model of order *p* implies that the current value of the time series is being predicted based on past *p*th data of the same random variable. Thus, an autoregressive model of order *p* can be expressed using the *p* previous values of the time series. Let *X*_1_, *X*_2_,…, be successive instances of the random variable *X*, measured at regular intervals of time. We applied the standard AR model and CA-AR (coercively adjusted coefficient with *φ* = 1 or −1) model to forecast the new *X*_*t*_ of the epilepsy EEG data. 

We forecasted the signals from 481 to 500 time points by the proposed model and compared the forecasting errors between the optimal order that decided by the average of fractal dimensions and the other orders. For forecasting, we used the *p* past values of time series, and *p* is selected by fractal dimension. If *p* = 5, our model uses from 476 to 480 time points to forecast 481 time point.  Table 1 shows RMSE results of several signals that were measured between the original values and the generated values by the model. RMSE is compared when the orders are 3, 5, 10, and 15 for AR and the proposed method. In the case of *p* = 5 in the proposed model, RMSE has a higher accuracy than *p* = 3, *p* = 10, and *p* = 15 in Subjects A, C, and E. In case of the standard AR, Subjects A, C and E exhibited the lowest RMSE in *p* = 5, similar to the proposed method. As shown in Table 1, the proposed method is exhibits a lower error rate than standard AR, and when *p* is 5, the lowest RMSE exhibited in Subject E among the subjects. Thus, we confirm that optimal order is 5, and it is used as the order of the CA-AR during experiments to verify the efficiency. The order of the proposed method is determined with the round-up integer of the average fractal dimension that it is measured from all normal signals as Subjects A and C.

**Figure 5:**
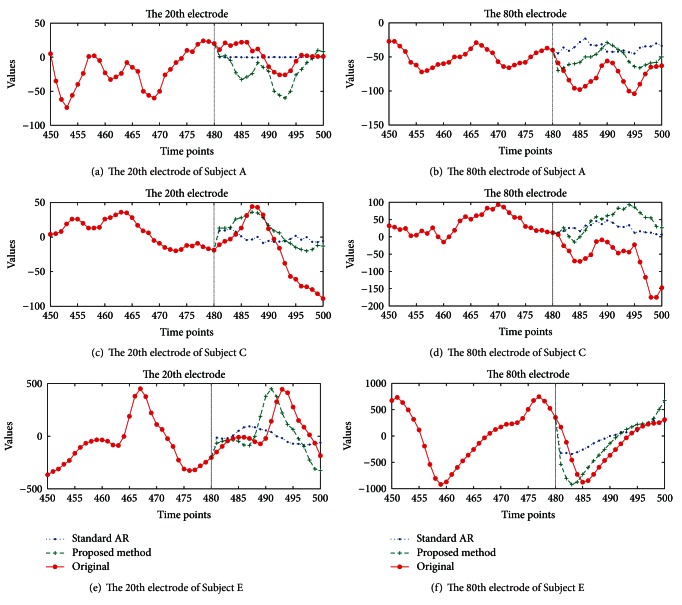
Forecast comparison: (a) and (b) plot forecasts of the 20th and 80th electrode of Subject A to compare the proposed and standard AR methods. (c) and (d) show forecast plots of Subject C. (e) and (f) display the forecast plot of the 20th and 80th electrode of Subject E, respectively.

Figure 5 shows the result of the forecast snapshot of Subjects A, C, and E, using the CA-AR and standard AR models. Plots (a), (c), and (e) show a specific case of a 20-time step prediction of the 20th electrode, and plots (b), (d), and (f) provide the prediction result for the 80th electrode signal. The original signal is shown in Figure 5(a), from the time 481 to 500 in red line with the star point marker. The plus sign marker shows the forecasted signals by the proposed method (green line) and the point marker plots the forecasted signals by standard AR (blue line). These plots confirm that our forecasting method outperforms the conventional AR method. 

In this paper, we compared the forecasting results among several existing methods and CA-AR method.  Table 2 shows the forecasting results using existing methods of linear and nonlinear prediction (Artificial Neural Networks [3], Fuzzy [2, 24], Nearest Neighbors [25], and the proposed method) of the 7th electrode signal in Subject E. For the experiments, we used a single signal of each subject to measure the forecasting error in each forecasting method. Also, to measure forecasting errors of existing methods, we derived the partial training signals from original signals that are from the starting points to 500 time points or 1000 time points of signal. For example, when the existing method tries to forecast the length of 150 time points, 1 to 500 time points or 1000 time point of the signal are used for training. However, our method still uses only 5 time points to forecast the future values of 150 time points. CA-AR considers from the time 496 to 500 to forecast the future values of the time 501, and it used from the time 497 to 501 to forecast the time 502. 

As a result of Table 2, the proposed method shows lower error rates than existing forecasting methods. In case of ANN, RMSE is gradually increased when the forecasting time points increase. However, our proposed method is unaffected by the length of the forecasting time points because it used only the order *p* of AR. Besides, even though the length of forecasting time points increased, the error rate of the forecasting result did not show much change in our model. In case of the Fuzzy-based method, it performs the batch process using all of the previous values to estimate the new value. Thus, we quit measuring the forecasting error because it requires very long execution time. 

Table 3 measures the forecasting time using the existing methods as in Table 2. For example, if the training time duration of ANN (Artificial Neural Networks) is 500 and the forecasting time duration is 150, then 0.5304 represents the execution time to forecast from the time 501 to 650. Fuzzy-based method [2, 24] was excluded from the 1500-forecasting time point test, because it already exhibited the highest forecasting time compared to the other methods in the 150-time point forecasting test. The proposed method achieves much faster forecasting time than ANN (Artificial Neural Networks) and NN (Nearest Neighbors). When the training time point or forecasting time point increases, the existing methods incrementally increase the forecasting time. However, since the proposed CA-AR method uses only a few observed values, it maintains a steady time. In the case of epilepsy seizure, we assume that it should be able to inform a patient a few minutes or several hours before the beginning of a seizure. 

We evaluated forecasting error with each signal in each subject, and Table 4 shows the results. This experiment is done to measure the future values of the length of 500 time points using the observed signal from the time 1 to 500. That is, single signal has 4096 time points, and the existing method used from the time 1 to 500 of single signal for learning. Models forecast from the time 501 to 1000 through learning. In this paper, we measured RMSE between the original signals and the forecasted signals. As a result of Table 4, Subjects C and E indicated the lowest average forecasting error rate when NN algorithm was used. However, NN method required much learning time compared to our method. That is, the values of 1 to 500 in NN will be the training period for the forecasted value of 501. For the forecast of 502, the training period is 1 : 501. On the other hand, our method provides the fast computation time because its training period is 407 to 501 for the forecast of 502. Besides, the difference of error rate between NN and CA-AR is very small.

Our method needs only the past values of *p* that are determined by fractal dimension to forecast the future values. Therefore, it guarantees the fastest computation time for learning to forecast the epileptic seizure. We measured the execution time of each model during forecasting the time length of 1000, 2000, and 3000. We can confirm the result of several signals from Table 5. In this experiment, we used each signal of Subject E and training signal used from the time 1 to 500 or 1000 time points of each signal in the existing method. As a result of Table 5, the lowest RMSE of forecasting is appeared in NN method except when CA-AR forecasted the length of the 2000 times using the length of the 1000 times as training signals. However, we need to look at the execution time of the whole forecasting method. NN method provided good forecasting results. However, it needs longer execution time. In addition, when the length of forecasting time increases, the execution time also increases. 

The accuracy of time series forecasting is a very important factor to many decision processes, and hence the research for improving the effectiveness of forecasting models has lasted. Both the neural network and the AR model capture all of the patterns in the data [26]. Our method also can capture the patterns of data because it was based on the AR model. In epilepsy EEG data, the amplitude between normal and seizure signal presents a great difference. If a pattern of the generated signals by the proposed model deviates from the past pattern, CA-AR can regard these as the epileptic seizure. However, the proposed model does not separately determine or measure the sliding time window length to detect the change of pattern in this paper. The goal of this study is to provide the fast run time and high forecasting accuracy in time series data that has special characteristics such as the nonperiodicity and non-linearity. Through our experiments results, we can guarantee the fast execution time and accuracy between original signal and generated signal from our model. 

## 4. Discussion

Epilepsy is a common neurological disorder in which some nerve cells spasmodically incur excessive electricity for a short time. Seizure predictions are mostly handled by statistical analysis methods from the EEG recordings of brain activity. The forecasting of epilepsy seizures can be used as a warning about seizures occurring on certain time scales by estimating the change in brain waves. That is, the forecasting of seizures alerts patients before an epilepsy seizure occurs. As a result, they could avoid potentially dangerous situations such as brain damage or injury during seizures.

In recent years, much research has looked into the prediction of epilepsy seizures using EEG data. Mormann et al. [27] analyzed bivariate EEG signals for seizure prediction. They analyzed the synchrony of EEG data using mean phase coherence (MPC) and maximum linear cross-correlation between EEG signal pairs. Schelter et al. [28] used MPC and obtained a proportion of seizures that were correctly predicted. Chávez et al. [29] analyzed the focal epilepsy EEG data for seizure prediction using non-linear regression analysis and phase synchrony. Winterhalder et al. [30] suggested the “seizure prediction characteristic” based on clinical and statistical considerations and compared to the performance of seizure prediction methods using concepts of linear and nonlinear time series analysis. This work indicates the uncertainty of predictions made by the use of the seizure occurrence period (SOP), in which the seizure is expected. However, it can be expressed when the independent variables are continuous. Moreover, these methods assume that the data has normality and independence. 

Li and Yao [31] proposed prediction methods based on the wavelet transform and fuzzy similarity measurements of EEG data. This method is divided into two steps: to calculate the entropy of the EEG data and to calculate similarity between variables. Li and Ouyang [32] proposed the dynamical similarity measure based on a similarity index to predict epileptic seizures using EEG data. Gigola et al. [33] analyzed the time domain of different types of epilepsy to predict epileptic seizures using wavelet analysis based on the evolution of accumulated energy. Maiwald et al. [34] evaluated three nonlinear methods for seizure prediction: dynamical similarity index, correlation dimension, and accumulated energy. These methods can extract robust features from EEG data. However, part of these methods is performed based on the window unit and it is not sufficient for clinical applications.

Several techniques have been proposed to analyze characteristics of seizures via various methods. Liu et al. [35] measured the fractal dimension of the human cerebellum in magnetic resonance images (MRI) of 24 healthy young subjects using the box-counting method. Esteller et al. [36] determined the fractal dimension in the cortex electroencephalogram (IEEG, ECoG), using the Katz algorithm. Their results show that an electrographic seizure in the Electrocorticography (ECoG) occurs when there is an increase of complexity. Sackellares et al. [37] found that temporal lobe epilepsy is characterized by episodic paroxysmal electrical discharges (ictal activity). These discharges consist of organized synchronous activity of mesial temporal neurons, particularly those of the hippocampus. However, proper interpretation of such analyses has not been thoroughly addressed. 

In this paper, we proposed a new CA-AR forecasting method based on the AR model that can forecast the seizure of complex epilepsy EEG data by applying the property of nonstandard distribution from [14]. The CA-AR model is suited to time series data with special characteristics, such as abnormality, noise, nonlinearity, and nonperiodicity. 

## 5. Conclusions

Epilepsy may be caused by a number of unrelated conditions, including damage resulting from high fever, stroke, toxicity, or electrolyte imbalances. An algorithm capable of effective real-time epileptic seizure prediction will allow the patient to take appropriate precautions minimizing the risk of a seizure attack or injuries resulting from such an attack. Conventional methods for forecasting or prediction of data require periodicity in the observed data. However, when we applied the cepstrum, seizure activity signals did not exhibit periodicity. In addition, we could distinguish whether the epilepsy EEG data is random or nonrandom using the lag plot. If the lag plot has a nonrandom pattern, it can be used for prediction by conventional approaches. However, our data appears to have a random distribution. 

This study proposed the random coefficients appropriate for random distribution data. Further, we used the log-log plots (box-counting) using the concept of fractal dimensions to forecast epilepsy EEG data to estimate the vital forecasting optimal order *p* in our CA-AR model. Our experimental results demonstrate that CA-AR (coercively adjusted autoregressive) is the most suitable forecasting method for nonperiodic data. It does not require complex calculations and conducts fast forecasting compared to other methods. In addition, our method generates future values more accurately or similar than other methods. The experiments on epilepsy EEG data show that our method is not only fast and scalable but also accurate in achieving low prediction errors. 

Future research could focus on extending CA-AR to perform forecasting on a multiple, coevolving time series which includes linear or non-linear correlations and periodicity or nonperiodicity. A more ambitious direction would be to automatically readjust the parameter and coefficient equations.

## Figures and Tables

**Table 1:** Root Mean Square Error of forecast using CA-AR and AR.

Electrode	Proposed method				Standard AR			
	*P* = 3	*P* = 5	*P* = 10	*P* = 15	*P* = 3	*P* = 5	*P* = 10	*P* = 15
RMSE of Subject A								

7	0.1878	0.1320	0.0419	0.0523	1.0044	0.8149	0.5294	0.5399
15	0.3766	0.5306	0.9532	0.9942	0.9947	0.9514	0.9985	1.3158
20	0.2629	0.1525	0.1943	0.3741	1.0003	1.0071	1.0478	1.0128
27	0.1226	0.0757	0.1347	0.0897	1.0093	1.0961	1.2423	1.3061
35	0.0213	0.0559	0.0348	0.0563	0.9995	0.9299	0.8068	0.8334
50	0.1271	0.1436	0.1093	0.1339	1.0038	0.9907	1.0183	1.1479
60	0.0356	0.0273	0.0332	0.0070	0.8364	0.5043	0.7012	0.5120
70	0.0554	0.0449	0.0369	0.0402	0.9960	0.9987	0.9937	1.0352
80	0.0101	0.0068	0.0078	0.0110	0.4783	0.3180	0.2287	0.2108
87	0.1368	0.1458	0.1524	0.1370	1.0011	0.9736	1.0224	1.3125
95	0.0999	0.0677	0.0902	0.0385	1.0025	1.0141	1.0118	0.9709

Average	0.1306	**0.1257**	0.1626	0.1758	0.9388	0.8726	0.8728	0.9270

RMSE of Subject C								

7	0.0302	0.0427	0.0372	0.0939	0.9501	0.5978	0.6480	0.6142
15	0.8090	0.7386	0.8873	1.5213	1.4048	1.1270	1.2222	1.8517
20	0.0384	0.0305	0.0335	0.0798	0.9979	0.9496	0.8857	0.8286
27	0.1288	0.1376	0.1122	0.1107	0.9469	1.02888	1.0845	0.7823
35	0.0495	0.0394	0.0428	0.1079	1.0635	1.0051	0.9715	0.9691
50	0.0369	0.0500	0.0437	0.0694	1.0187	0.9474	0.9118	0.8815
60	0.0319	0.0412	0.0282	0.1105	0.9208	0.6429	0.7676	0.7703
70	0.1612	0.1302	0.1527	0.0376	1.0973	1.3006	1.2392	1.3000
80	0.1068	0.1126	0.1087	0.1012	1.2428	1.4660	1.4313	1.4892
87	0.1478	0.1470	0.1621	0.0706	1.1839	1.5971	1.6903	1.9786
95	0.1158	0.1105	0.1141	0.0710	1.6958	1.7924	1.6513	1.6922

Average	0.1506	**0.1437**	0.1566	0.2158	1.1384	1.1322	1.1367	1.1962

RMSE of Subject E								

7	0.2225	0.0098	0.0074	0.0272	0.9566	0.9761	0.7667	0.7669
15	0.0709	0.0749	0.0763	0.0739	1.0041	0.9226	0.9306	0.9534
20	0.1757	0.0540	0.1248	0.1429	1.2710	1.1595	0.9591	0.3047
27	0.0661	0.0494	0.0822	0.1094	1.1139	0.9623	1.1128	0.8602
35	0.1138	0.0375	0.0605	0.0774	1.0139	1.0181	1.2581	2.8540
50	0.0345	0.0310	0.0121	0.1890	0.7694	0.8749	0.8942	1.6892
60	0.0062	0.0343	0.0109	0.0794	0.8563	0.6543	0.8975	1.0238
70	0.0975	0.0246	0.0590	0.0450	0.1912	1.1594	1.7443	1.5908
80	0.0642	0.0237	0.0517	0.1455	0.5931	0.5950	3.4392	9.2907
87	0.0039	0.0802	0.1155	0.1507	1.0098	0.5522	0.5753	0.6716
95	0.1567	0.0389	0.1023	0.1285	1.2754	1.1505	1.0432	0.619

Average	0.0920	**0.0417**	0.0639	0.1063	0.9141	0.9114	1.2383	1.8749

**Table 2:** Root Mean Square Error of forecast comparison.

Forecasting time points	500 (training time points)				1000 (training time points)			
	ANN	Fuzzy	NN	CA-AR (5)	ANN	Fuzzy	NN	CA-AR (5)
150	0.2059	0.1210	0.1462	0.0162	0.1297	0.0721	0.0950	0.0114
1500	0.6174	—	0.1050	0.0541	0.3221	—	0.0887	0.0533
2000	0.6328	—	0.0998	0.0487	0.356	—	0.0918	0.0486
2500	0.7607	—	0.0995	0.0497	0.3741	—	0.0910	0.0507
3000	0.7801	—	0.0976	0.0480	0.3613	—	0.0874	0.0479

**Table 3:** Forecast time comparisons.

Forecasting time points	500 (training time points)				1000 (training time points)			
	ANN	Fuzzy	NN	CA-AR (5)	ANN	Fuzzy	NN	CA-AR (5)
150	0.5304	186.94	1.060	0.0780	4.430	675.38	1.669	0.0156
1500	0.9516	—	13.722	0.0499	5.004	—	13.887	0.0811
2000	0.9953	—	20.439	0.0749	4.995	—	20.689	0.0718
2500	0.9766	—	28.189	0.0967	5.098	—	29.178	0.0874
3000	1.0764	—	32.723	0.0748	5.248	—	37.272	0.0736

**Table 4:** The measured forecasting error with several signals from each subject.

Electrode	Subject A				Subject C				Subject E			
	NN	Fuzzy	ANN	CA-AR (5)	NN	Fuzzy	ANN	CA-AR (5)	NN	Fuzzy	ANN	CA-AR (5)
4	0.320	0.050	0.429	0.019	0.013	0.031	0.156	0.023	0.018	0.044	0.136	0.029
8	0.086	0.050	0.177	0.127	0.010	0.018	0.093	0.018	0.002	0.121	0.086	0.016
35	0.043	0.086	0.113	0.056	0.045	0.029	0.394	0.078	0.069	0.169	0.203	0.038
70	0.145	0.058	0.166	0.045	0.038	0.048	0.130	0.066	0.012	0.072	0.038	0.025
95	0.093	0.087	0.173	0.068	0.030	0.061	0.111	0.024	0.013	0.081	0.115	0.039

Average	0.137	0.066	0.212	**0.063**	**0.027**	0.037	0.177	0.042	**0.023**	0.097	0.115	0.029

**Table 5:** Comparison of the foresting error rates and the execution time between the existing methods and the proposed method.

Forecasting time points	Electrodes	RMSE						Execution time (sec)					
		500 (training)			1000 (training)			500 (training)			1000 (training)		
		ANN	NN	CA-AR (5)	ANN	NN	CA-AR (5)	ANN	NN	CA-AR (5)	ANN	NN	CA-AR (5)
1000	4	0.091	0.020	0.047	0.13	0.023	0.05	1.01	7.44	0.08	5.34	9.11	0.06
	8	0.039	0.006	0.043	0.055	0.016	0.051	0.08	7.75	0.03	5.01	8.86	0.06
	35	0.228	0.072	0.039	0.192	0.062	0.045	0.90	7.64	0.08	4.90	8.78	0.05
	70	0.057	0.010	0.056	0.049	0.011	0.054	0.10	7.57	0.08	4.48	8.63	0.06
	95	0.091	0.032	0.044	0.145	0.038	0.034	0.94	7.44	0.06	4.70	8.75	0.05

	Average	0.101	**0.028**	0.046	0.114	**0.03**	0.047	0.61	7.57	**0.07**	4.88	8.83	**0.06**

2000	4	0.217	0.021	0.046	0.245	0.03	0.038	1.11	20.55	0.08	4.87	20.14	0.05
	8	0.060	0.019	0.047	0.082	0.027	0.018	1.11	20.64	0.08	5.55	20.87	0.05
	35	0.253	0.063	0.052	0.191	0.088	0.048	0.83	20.31	0.06	4.91	21.03	0.05
	70	0.058	0.015	0.049	0.046	0.012	0.033	1.01	20.12	0.08	4.52	20.94	0.04
	95	0.173	0.037	0.048	0.198	0.038	0.048	0.92	20.58	0.08	5.12	20.47	0.05

	Average	0.152	**0.031**	0.048	0.152	0.039	**0.037**	1.00	20.44	**0.07**	5.00	20.69	**0.05**

3000	4	0.376	0.029	0.052	0.399	0.019	0.051	0.92	32.74	0.08	5.79	38.31	0.05
	8	0.094	0.015	0.051	0.112	0.032	0.051	1.22	32.39	0.03	5.68	36.47	0.05
	35	0.259	0.070	0.049	0.213	0.087	0.047	0.98	32.40	0.08	4.76	36.57	0.05
	70	0.055	0.023	0.048	0.044	0.013	0.046	1.33	32.79	0.08	5.13	37.53	0.05
	95	0.227	0.049	0.050	0.233	0.059	0.049	0.94	33.29	0.11	4.88	37.47	0.05

	Average	0.202	**0.037**	0.050	0.2	**0.042**	0.049	1.08	32.72	**0.07**	5.25	37.27	**0.05**
